# Most Common Pathway of Metastasis of Rectal Signet Ring Cell Carcinoma to the Skin: Hematogenous

**DOI:** 10.7759/cureus.6890

**Published:** 2020-02-05

**Authors:** Bayarmaa Mandzhieva, Anum Jalil, Mahum Nadeem, Syed Askari Hasan, Akriti G Jain

**Affiliations:** 1 Internal Medicine, AdventHealth, Orlando, USA; 2 Internal Medicine, University of Oklahoma, Oklahoma City, USA; 3 Internal Medicine, Florida Hospital, Orlando, USA

**Keywords:** cutaneous metastasis, signet ring cell, colorectal cancer, hematogenous, rectal stump

## Abstract

Liver represents the most common site of metastasis in patients with advanced colorectal cancer (CRC). Cutaneous metastasis is uncommon and has been documented only in 3% of patients. Most cutaneous metastases demonstrate typical histological features of adenocarcinoma, such as glandular formation and mucin production. We present the case of a 66-year-old male with Crohn’s disease (CD) and stage IV rectal signet ring cell carcinoma arising in his rectal stump who presented with a painful papular bilateral groin rash. Biopsy revealed metastatic signet ring cell carcinoma. Since cutaneous metastasis in patients with advanced CRC can be easily confused with infection, especially fungal infection, physicians should be vigilant of the possibility of cutaneous metastasis. Our literature review suggests hematogenous spread as the dissemination pathway of this histological subtype of rectal adenocarcinoma to the skin. We present the first case of cutaneous metastatic signet ring cell carcinoma from a rectal stump of a patient with CD.

## Introduction

Cutaneous metastasis is relatively rare in colorectal cancer (CRC). It has been reported that skin metastasis usually develops within two years after resection of the primary tumor [1-3]. Such metastasis indicates advanced stage and associated with poor prognosis due to suboptimal treatment and aggressive biological behavior of tumor.

Cutaneous metastatic lesions can present with a variety of morphological appearances, but histological features generally mimic the primary cancer. The most frequent site is the abdomen with an operative scar while scrotum being an uncommon site. It is crucial for clinicians to be aware of skin metastasis in patients with a CRC history who present with cutaneous lesions. Early biopsy may be a reasonable approach.

Our case documents the first incidence of cutaneous metastatic signet ring cell carcinoma from a rectal stump of a patient with Crohn’s disease (CD) which presented as a painful papular rash in his groins and scrotum. 

## Case presentation

**Figure 1:**
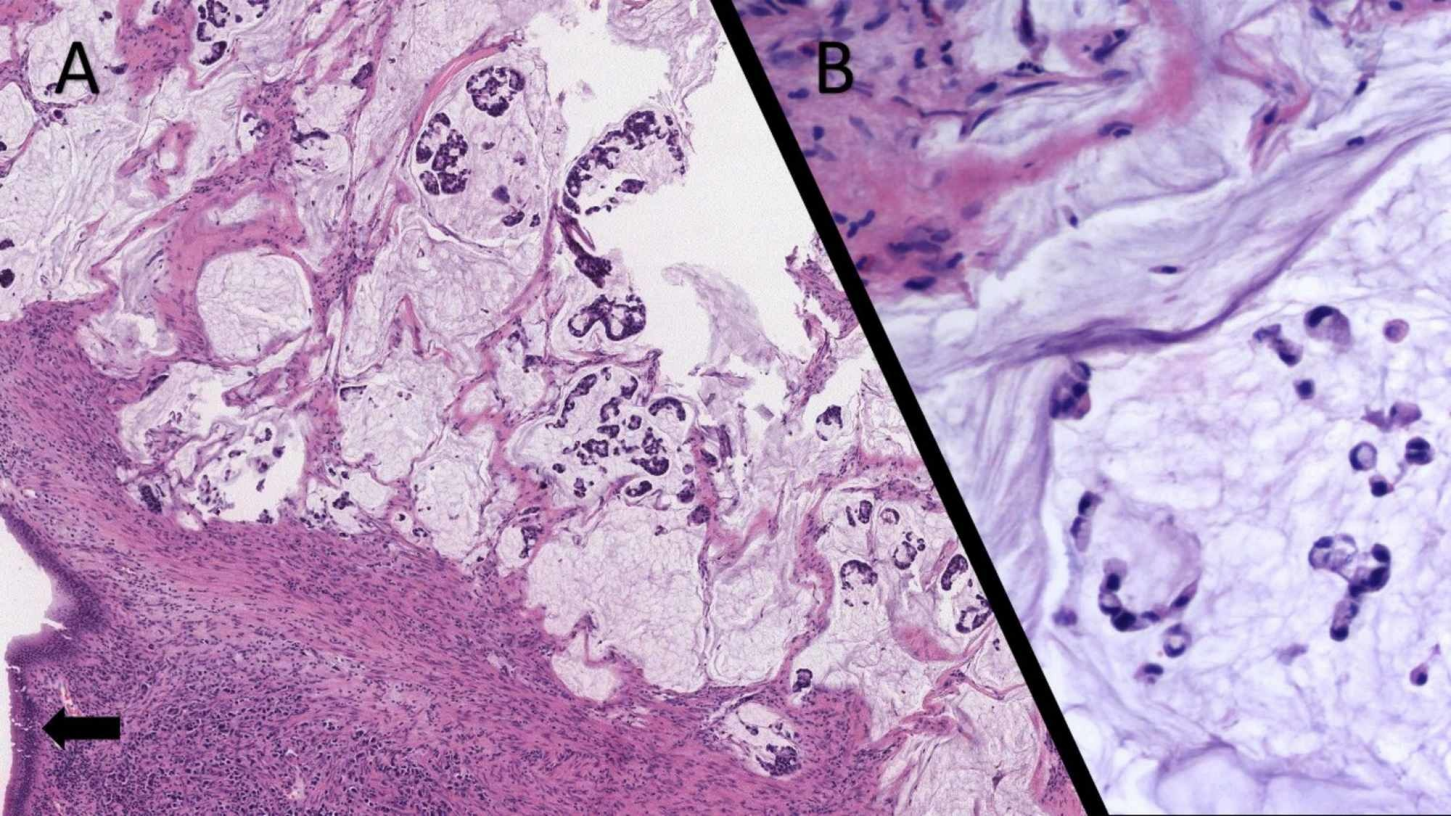
Rectal Signet Ring Cell Carcinoma (A) A section of the rectal stump shows a mucin-producing adenocarcinoma invading the rectal wall. The black arrow indicates the rectal mucosa. (B) A high-power view reveals many neoplastic cells with a typical signet ring cell appearance characterized by abundant intracytoplasmic mucin, displacing the nucleus to one side of the cell.

The patient was a 66-year-old male with a history of CD and past surgical history of total colectomy with end ileostomy. He presented with rectal pain that could not be controlled with conservative treatments, and therefore underwent a complete proctectomy. The histological examination of the resected rectal stump unexpectedly revealed a signet ring cell adenocarcinoma (Figure 1A, 1B) extending through the full thickness of the colonic wall to invade the pericolic soft tissue. Three pericolic lymph nodes were positive for metastatic signet ring cell adenocarcinoma for a pathological stage of PT3N1b. Subsequently, the patient underwent radiation therapy and chemotherapy with FOLFOX (leucovorin, 5-fluorouracil, and oxaliplatin). 

**Figure 2:**
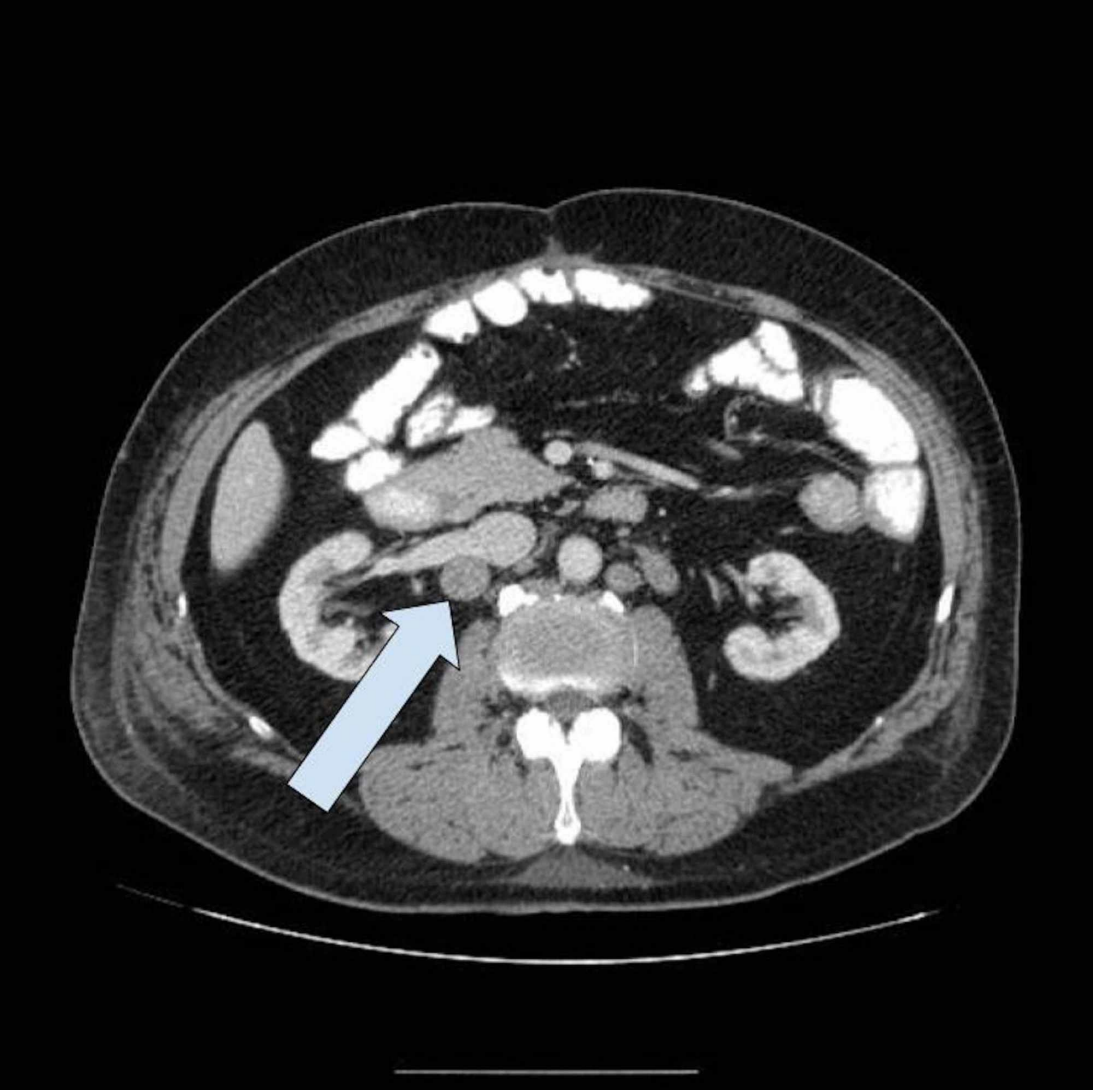
CT Abdomen and Pelvis with IV Contrast Interval worsening of retroperitoneal lymphadenopathy, representing metastatic rectal cancer.

**Figure 3:**
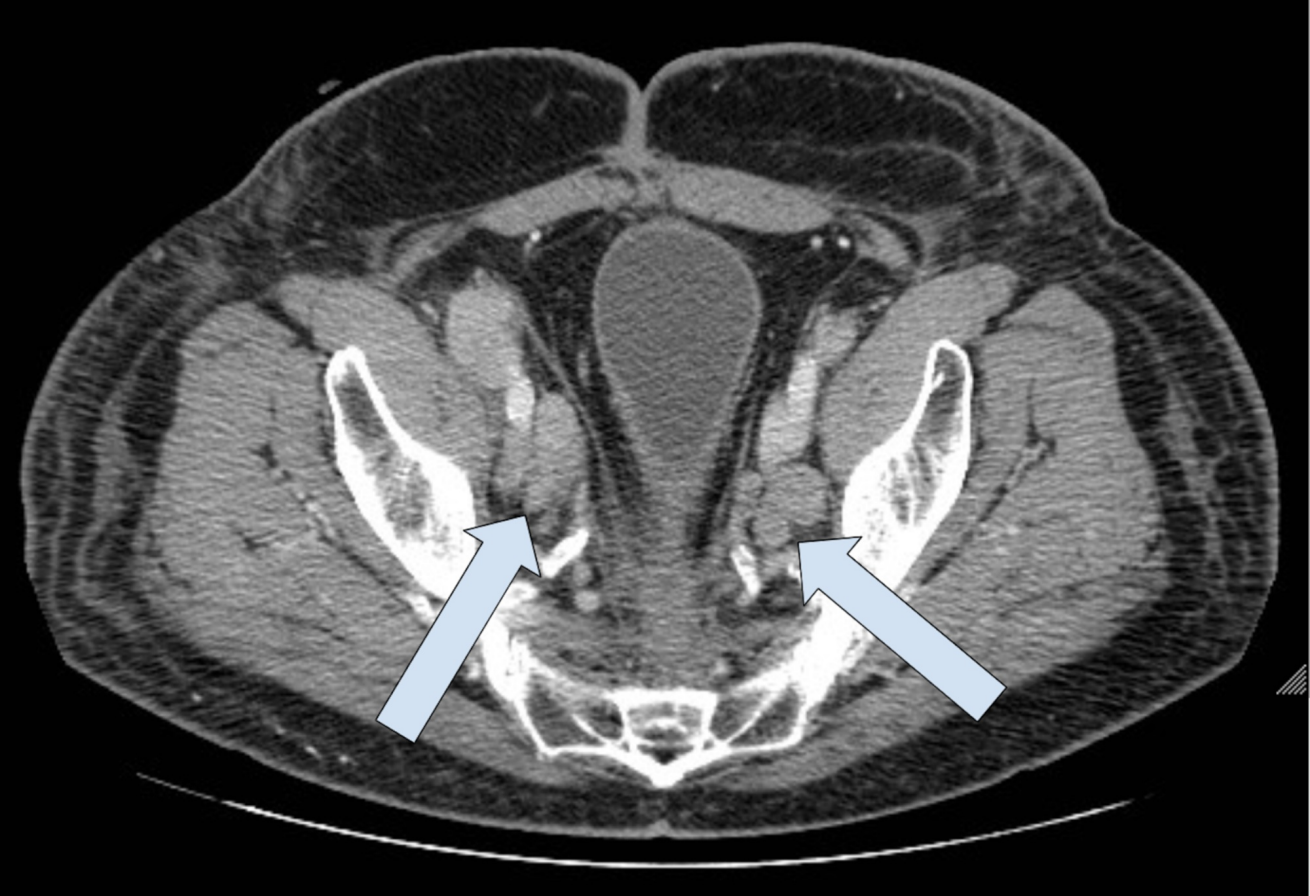
CT Abdomen and Pelvis with IV Contrast Interval worsening of pelvic sidewall lymphadenopathy, representing metastatic rectal cancer.

**Figure 4:**
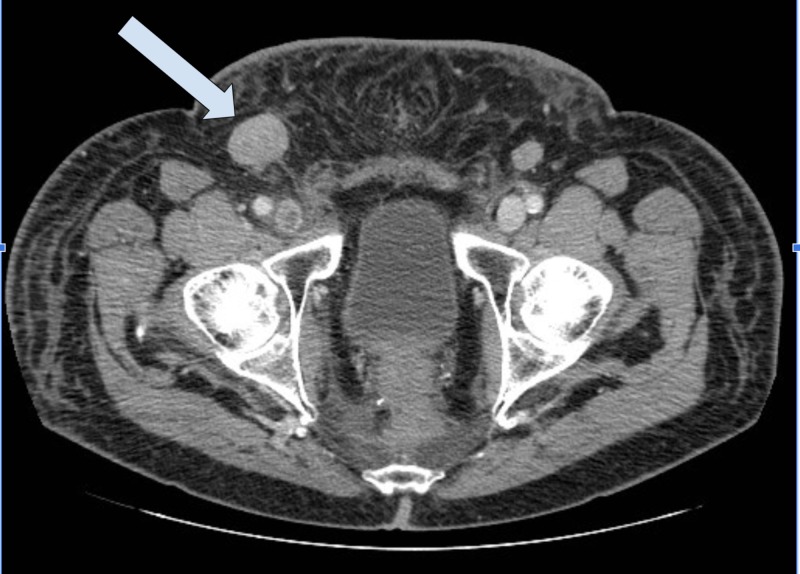
CT Abdomen and Pelvis with IV Contrast Interval worsening of inguinal lymphadenopathy, representing metastatic rectal cancer.

Five months post proctectomy, the patient was found to have extensive retroperitoneal (Figure 2), pelvic (Figure 3), and inguinal lymphadenopathy (Figure 4), consistent with nodal metastasis from the rectal carcinoma.

**Figure 5:**
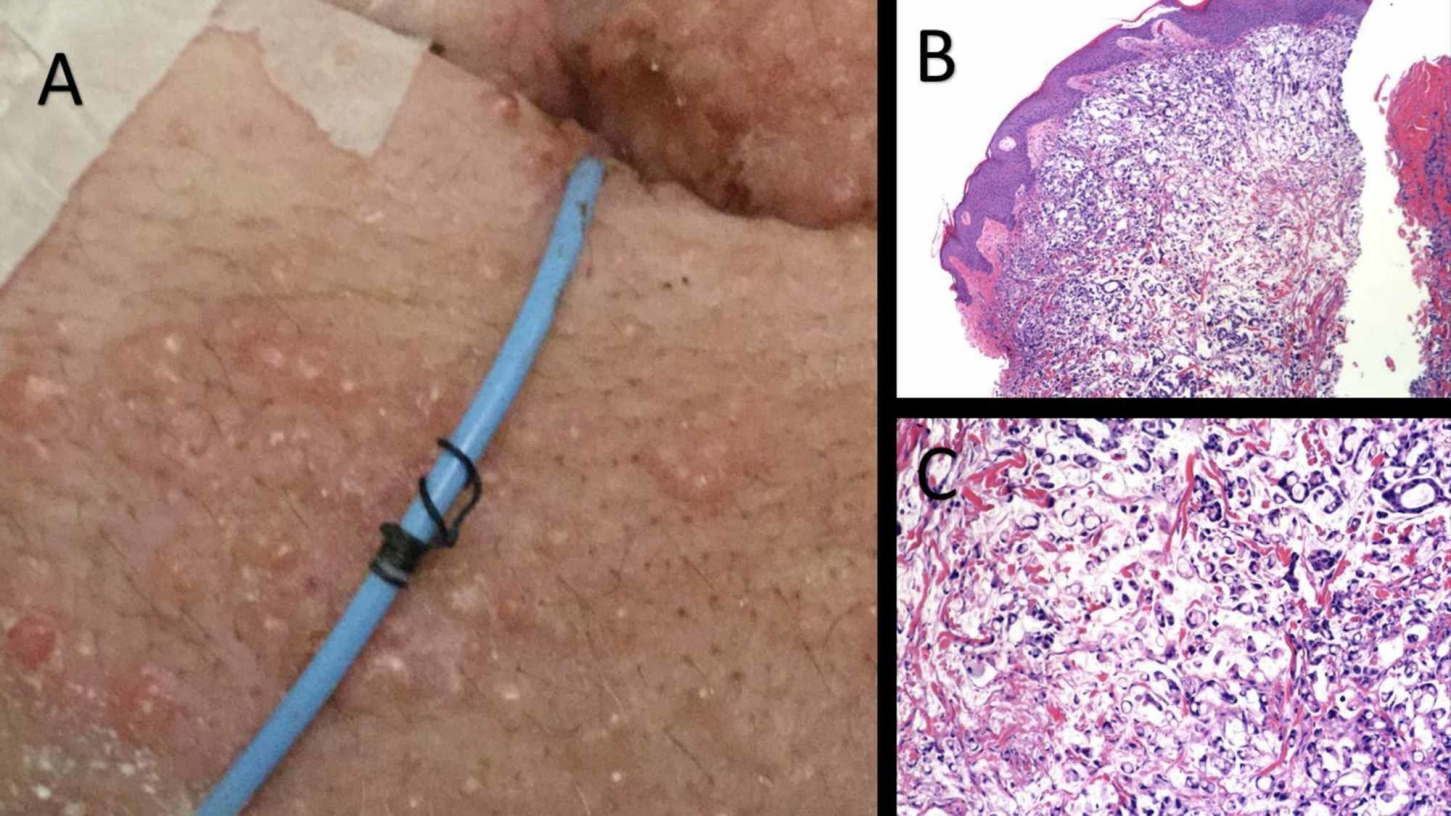
Cutaneous Metastasis with Histopathology and Lymph Node Biopsy Bilateral groin papular skin rash with several small papules, some with a vesicular appearance (A). A biopsy reveals a mucin-producing adenocarcinoma in the dermis (B) composed of many signet ring tumor cells. Core biopsy of a right inguinal lymph node also revealed nodal metastatic signet ring cell carcinoma (C).

Three months later, he presented with bilateral groin rash of several weeks. At admission, several non-pruritic painful papules were present in his groins and scrotum (Figure 5A). Some papules appeared vesicular, which raised a spectrum of clinical differential diagnoses. During the hospital stay, the groin rash began to spread to the suprapubic area and the ostomy site, increased in size, and a few lesions became umbilicated. A punch biopsy showed sheets of signet ring cells in a mucinous background involving the dermis (Figure 5B). In addition, a core biopsy of a right inguinal lymph node also revealed nodal metastatic signet ring cell carcinoma (Figure 5C). Therefore, six rounds of palliative radiotherapy were given. Unfortunately, subsequent contrasted tomography of abdomen revealed progression of the disease and the patient decided to opt for hospice.

 

## Discussion

Cutaneous metastases of internal malignancies are rare with only 1.3% primary tumors display cutaneous manifestations at the time of diagnosis [2]. According to Hu et al., the most common cancer known to spread to the skin in women is breast cancer with an incidence of 24% and melanoma and lung cancer in males [4]. Other types of malignancies including lung, colorectal, renal, ovarian, and bladder have similar rates of cutaneous metastasis, which vary from 3.4% to 4.0% [4]. 

Only 3% of CRCs metastasize to the skin. The abdomen, particularly at surgical incision scars (both ad hoc surgical scar and preexisting unrelated operative scars), is the most frequent site of cutaneous metastasis of colonic cancer with the pelvis, back, chest, upper extremities, head and neck being the less common ones. This may occur through lymphatic spread, intravascular dissemination, direct extension of tumor, surgical implantation, or spread along embryonal remnants such as the urachus [5].

Clinical features can vary from flesh colored to violaceous, freely mobile to fixed, single to multiple painless to painful nodules, ulcer, bullae, or fibrotic processes [5]. Apart from renal cell carcinoma with its typical histological features, cutaneous metastases do not always permit identification of the primary tumor and immunohistochemical studies are usually required to confirm the primary site [6]. 

According to the literature, most cases of cutaneous metastasis composed of signet ring cells are of gastric origin. On the other hand, metastatic signet ring cell carcinoma of colorectal origin to the skin is rare. In a study of cutaneous metastases in patients with CRC, Atalay and Yılmaz found eight cases of cutaneous metastasis in a cohort of 263 patients (3%) [7]. Their histopathological analysis revealed only typical and mucinous adenocarcinoma at the site of cutaneous metastasis. No case of metastatic signet ring cell carcinoma to the skin was observed in any of their patients. In another study of six patients with cutaneous metastases of rectosigmoid origin, all cutaneous metastases displayed the typical adenocarcinoma without any signet ring cell case [8]. In a literature review of 28 cases of cutaneous metastases adenocarcinoma from the rectum, Dehal et al. identified only two definitive cases of rectal signal ring cell adenocarcinoma metastasizing to the skin [9]. Our own literature review via the PubMed search engine revealed only five histologically well-documented cases of metastatic signet ring cell carcinoma of rectal origin to the skin (Table 1) [10-14].

**Table 1:** Cases Rectal Signet Ring Cell Adenocarcinoma with Cutaneous Metastases M: male, F: female, pos/neg. lns: positive/negative numbers of lymph nodes, m.: months; DOD, dead of disease.

Case	Gender/age	Stage (pos/neg. lns)	Other organs	Elapse time/number	Site	Size	Symptoms and histology	Follow-up
Tsai et al. [10]	45M	pT4N1M0 (9/10)	Lung (15 m.)	11 m., multiple	Shoulder, face, neck, scalp, back, abdomen	N/A	N/A	DOD 4 m. after skin
Tan et al. [11]	70M	pT3N2M0 (6/15)	None	22 m., x1	Left scapula	10 cm	Discomfort, dermal and subcutaneous	Wide excision and further palliative chemo
Kilickap et al. [12]	29M	Stage III	Liver (18 m.)	14 m., x2	Chest wall and left midaxillary line	1 x 2 cm	Painless subcutaneous	Chemotherapy
Moonda et al. [13]	71M	N3 (21/23)	None	12 m., x1	Upper lip	1.5 cm	Dermis	Lost to follow-up
Rodrigues et al. [14]	38F	T4b	Pelvic and retroperitoneal lymphadenopathy Peritoneal carcinomatosis	2 m, x2	Right costal margin	1cm	Hard nodules subcutaneous	DOD (4 m. from skin diagnosis)
Mandzhieva et al. (2019)	66M	pT3N1b (3/3)	Retroperitoneal, pelvic, and inguinal lymphadenopathy	6 m., multiple	Bilateral groin, pubic, scrotum	0.3-0.5 cm	Dermis	Hospice (2 m. from skin diagnosis)

Further analysis of these cases revealed several additional interesting observations, including most patients were male (5:1) with a mean age of 53 years (range: 29-71 years). Most cutaneous metastases were clinically detected in less than one year (mean: 11 months, range: 2-22 months). The detection of cutaneous metastases portends a dismal prognosis with most patients dying in less than six months (2-4 months). When cutaneous metastases were seen, distant metastases were not documented in the liver or lungs of any patients although liver and pulmonary metastases were reported four months after the skin metastases in two patients [10,12]. Cutaneous metastases of rectal signet ring cell carcinoma displayed an atypical skin distribution with a high incidence on the face and upper body in most of the cases. 

It is hypothesized that “if tumor cells invade vessels, they present as cutaneous metastasis at distant sites, while if they involve lymphatics, late local recurrence at a primary site is common” [15]. The absence of concomitant liver metastases at the time of diagnosis of cutaneous metastases in patients with signet ring cell carcinoma (Table 1) did not argue against a hematogenous dissemination of the tumor cells as blood in the mid and lower rectum is drained predominantly through the rectal venous plexus into the inferior venacava and bypassing the portal vein and therefore the liver [16]. Another possibility is that the metastases in the liver are micrometastases due to the short time elapse between the diagnosis of the rectal signet ring cell carcinoma and the cutaneous metastases, and therefore are undetectable by radiological studies at the time that cutaneous metastases are detected [15]. Survival after diagnosis of cutaneous metastasis can range from one month to three years, with average survival around 18 months [17]. 

## Conclusions

Cutaneous metastasis of CRC is rare, but well described in the literature. It usually indicates widespread disease and poor prognosis. Hence, physicians should be vigilant about careful examination of the skin even after a long asymptomatic period. High index of suspicion should be maintained and biopsy performed in a patient with CRC history, particularly for unexplained skin nodules.
